# Subcutaneous Tissue: To Suture or Not to Suture at Cesarean Section

**DOI:** 10.1155/S1064744994000219

**Published:** 1994

**Authors:** Van R. Bohman, Larry C. Gilstrap III, Susan M. Ramin, Bertis B. Little, Rigoberto Santos-Ramos, Kenneth G. Goldaber, Jody Dax, Kenneth J. Leveno

**Affiliations:** Department of Obstetrics and Gynecology University of Texas Southwestern Medical School 5323 Harry Hines Boulevard Dallas TX 75235-9032 USA

## Abstract

*Objective:* The null hypothesis for this investigation was that there was no difference in the frequency of wound disruption between women who had their subcutaneous tissues approximated with suture and those who did not during cesarean section.

*Methods:* During alternating months, consecutive women delivered by cesarean section either did (N = 716) or did not (N = 693) have their subcutaneous tissues closed with suture. All data were analyzed using chi square, Student's t-test, Fisher's exact probability test, analysis of variance, or logistic regression.

*Results:* A 32% decrease in the frequency of wound disruption was observed when subcutaneous tissues were brought into apposition with suture at cesarean section (*P* = 0.03).

*Conclusions:* Closure of Scarpa's and Camper's fascia with suture during cesarean section significantly decreased the frequency of wound disruption in this population.


Infectious Diseases in Obstetrics and Gynecology 1:259–264 (1994)
© 1994 Wiley-Liss, Inc.

### Subcutaneous Tissue: To Suture or Not to Suture at Cesarean Section

**Van R. Bohman, Larry C. Gilstrap III, Susan M. Ramin, Bertis B. Little, Rigoberto Santos-Ramos, Kenneth G. Goldaber, Jody Dax, and Kenneth J. Leveno**

*Department of Obstetrics and Gynecology, University of Texas Southwestern Medical School, Dallas, TX*

ABSTRACT

***Objective:*** **The null hypothesis for this investigation was that there was no difference in the frequency of wound disruption between women who had their subcutaneous tissues approximated with suture and those who did not during cesarean section.**

***Methods:*** **During alternating months, consecutive women delivered by cesarean section either did (N = 716) or did not (N = 693) have their subcutaneous tissues closed with suture. All data were analyzed using chi square, Student's t-test, Fisher's exact probability test, analysis of variance, or logistic regression.**

***Results:*** **A 32% decrease in the frequency of wound disruption was observed when subcutaneous tissues were brought into apposition with suture at cesarean section ($P = 0.03$).**

***Conclusions:*** **Closure of Scarpa's and Camper's fascia with suture during cesarean section significantly decreased the frequency of wound disruption in this population.** © 1994 Wiley-Liss, Inc.

KEY WORDS

Wound disruption, infection, wound closure

Approximately 1 in 4 women in the United States are delivered by cesarean section, and it is well established that operative abdominal delivery is associated with a significant risk of infection compared with vaginal delivery. Another major complication is wound disruption, which may be associated with infections, hematomas, seromas, pulmonic complications, host immunocompromise, and poor nutritional status.[1–12] These risks are increased with preexisting operative site infection, breaks in sterile technique,[7] prolonged preoperative admissions[10] that may result in colonization with resistant microbes, prolonged operative duration,[2,8] use of electrocautery,[8] obesity,[3,9] advanced age, inadequate host immunocompetence,[10] increased abdominal pressure,[9] liver disease, malnutrition, steroid use, malignancies,[8] and introduction of foreign material or devitalized tissues to the healing site.[7,11] Wound breakdown leads to a prolonged hospital stay with added discomfort and may add significantly to the already overburdened health care dollar.

There is no consensus of opinion regarding whether or not it is advantageous to approximate Scarpa's and Camper's fascia with suture ligatures. Approximating these tissue layers with suture obliterates dead space and helps approximate the skin edges, but also adds foreign material to the healing site. The purpose of the present investigation was to compare the frequency of wound disruption between cesarean section wounds that did and did not have Scarpa's and Camper's fascia approximated with suture.

Address correspondence/reprint requests to Dr. Larry C. Gilstrap III, Department of Obstetrics and Gynecology, University of Texas Southwestern Medical School, 5323 Harry Hines Boulevard, Dallas, TX 75235-9032.

Received December 21, 1993

Accepted April 25, 1994

Clinical Study

### SUBJECTS AND METHODS

All pregnancies delivered by cesarean section at Parkland Memorial Hospital from April 1, 1991, through September 30, 1991, were assigned, on an alternating-month basis, to have Scarpa's and Camper's fascia closed or not closed with interrupted 000 plain catgut suture. Either a vertical or transverse (pfannenstiel) skin incision was utilized. All uterine incisions were closed with #1 chromic suture, bladder flaps with 00 chromic suture, parietal peritoneum with 00 chromic suture, and rectus fascia with 0 polydioxanone suture. The skin edges were approximated with surgical staples (Visi Stat skin stapler, Edward Weck & Company, Inc., Research Triangle Park, NC). Staples were removed 3–5 days after the operation, and the operative site was secured with adhesive tapes (shurstrips, Inman Leibelt Corporation, Arlington, TX).

Amnionitis was defined as labor complicated by maternal fever (≥38°C) or an infant with a foul odor at delivery. Puerperal endometritis was defined as a fever (≥38°C) with uterine and parametrial tenderness. The Quetelet's index, a derived value that represents weight/unit height, was calculated with the following formula: weight (kg)/ height$^2$ (m).[13] Obesity was defined as a Quetelet's index value >25.

Superficial wound disruption was diagnosed when tissues above the rectus fascia did not remain in apposition during the puerperal period and involved the entire length of the incision. Fascial dehiscence was diagnosed when the integrity of the rectus fascia was lost. Operative time longer than 1 h was considered prolonged. Excessive blood loss was defined as a 10-volume % decrease in the hematocrit between admission and the 1st postoperative morning. Perioperative antimicrobial prophylaxis was used and typically included cefazolin, cefotetan, or ampicillin/sulbactam. Prolonged rupture of membranes was defined as longer than 24 h.

Each patient's clinical history and progress were reviewed, computerized, and statistically analyzed utilizing chi square, Fisher's exact probability test, Student's t-test, analysis of variance, and logistic regression models using SAS software (SAS Institute, Inc., Cary, NC). Probabilities ≤0.05 were considered significant.

### RESULTS

A total of 7,670 women were delivered during the study period and 1,428 (18.6%) received cesarean sections. Nineteen women with cesarean sections were excluded because of incomplete data (N = 15), or laparotomy was performed for indications other than cesarean section (N = 4). In the remaining 1,409 pregnancies, 1,184 (84%) had a vertical skin incision and 225 (16%) had a low transverse, pfannenstiel-type incision. Six hundred seven (51%) of the patients with a vertical incision and 109 (48%) of those with a pfannenstiel incision had closure of the subcutaneous tissue. There were no significant differences in demographic characteristics between the suture and non-suture groups (Table 1) or vertical vs. transverse skin incision. Skin staples were used to approximate the skin edges in 1,369 (97%) patients.

Selected pregnancy outcomes are summarized in Table 2. The relative frequency of indications for these cesarean sections and type of skin incision did not differ between the 2 study groups. Similarly, the 2 study groups were comparable with respect to prolonged operative time, excessive blood loss, puerperal infection, and fascial dehiscence. However, although not significant, rectus fascia dehiscence (N = 3) was limited to the non-subcutaneous suture group. Superficial wound disruption occurred in a total of 96 (7%) women. Superficial wound disruption was significantly decreased in those women with suture approximation of Scarpa's and Camper's fascia prior to stapling the skin. Specifically, 59 (9%) of the non-suture closures disrupted compared with 40 (6%, $P = 0.03$) women with sutures placed into Scarpa's and Camper's fascia. Ninety-one (7.7%) of 1,184 with a vertical skin incision vs. 5 (2.2%) of 225 with a pfannenstiel incision ($P = 0.004$) had a superficial wound dehiscence.

The incidence of superficial wound disruption was not significantly affected by the choice of antimicrobial utilized for prophylaxis. Four hundred forty-nine women received a single 2 g intravenous (IV) dose of cefazolin for prophylaxis and had a wound disruption rate of 7.3% (N = 33); 447 received a single 2 g IV dose of cefotetan and had a wound disruption rate of 5.3% (N = 24); and 177 received a single IV dose of 2 g ampicillin/1 g sulbactam and had a wound disruption rate of 8.5% (N = 15).

TABLE 1. Pregnancy demographics in 1,409 women with and without subcutaneous sutures at cesarean section[a]

| Demographic factor | Subcutaneous suture Yes (N = 716) | Subcutaneous suture No (N = 693) | Comparison |
|---|---|---|---|
| Age (years) | 25 ± 6[b] | 25 ± 6[b] | NS |
| Gravidity | 3 ± 2 | 3 ± 2 | NS |
| Parity | 2 ± 1 | 2 ± 1 | NS |
| Obesity | 29 ± 7 | 29 ± 6 | NS |
| Operative time (min) | 48 ± 18 | 49 ± 19 | NS |
| Rupture of membranes (h) | 5 ± 14 | 6 ± 28 | NS |
| Race | | | |
| Hispanic | 331 (46%)[c] | 301 (43%)[c] | NS |
| Black | 215 (30%) | 235 (34%) | NS |
| White | 153 (21%) | 141 (20%) | NS |
| Other | 17 (2%) | 16 (2%) | NS |
| Labor | 416 (58%) | 403 (58%) | NS |
| Prolonged rupture of membranes | 93 (13%) | 100 (14%) | NS |
| Diabetes | 34 (4%) | 21 (3%) | NS |
| Amnionitis | 52 (7%) | 58 (8%) | NS |

[a]Differences in the mean compared with Student's t-test; differences in the frequency compared with chi square; obesity = Quetelet's index value >25 [weight (kg)/height$^2$ (m)]; prolonged rupture of membranes = >24 h; amnionitis = fever ≥38°C in labor and/or foul-smelling infant at delivery; NS = not significant.
[b]Mean ± SD.
[c]Number with percentage in parentheses.

TABLE 2. Pregnancy outcomes in 1,409 women with and without subcutaneous sutures at cesarean section[a]

| Outcome | Subcutaneous suture Yes (N = 716) | Subcutaneous suture No (N = 693) | Comparison |
|---|---|---|---|
| Indication for cesarean section | | | |
| Repeat | 326 (46%)[b] | 300 (43%)[b] | NS |
| Dystocia | 166 (23%) | 177 (26%) | NS |
| Abnormal presentation | 115 (16%) | 96 (14%) | NS |
| Fetal distress | 95 (13%) | 105 (15%) | NS |
| Other | 14 (2%) | 15 (2%) | NS |
| Skin incision type | | | |
| Pfannenstiel | 109 (15%) | 116 (17%) | NS |
| Vertical | 607 (85%) | 577 (83%) | NS |
| Prolonged operative time | 109 (15%) | 118 (17%) | NS |
| Excessive blood loss | 32 (5%) | 34 (5%) | NS |
| Endometritis | 166 (23%) | 169 (24%) | NS |
| Fascial dehiscence | 0 (0%) | 3 (0.4%) | NS[c] |
| Superficial wound disruption | 40 (6%) | 59 (9%) | 0.03 |

[a]Frequencies compared with chi square; prolonged operative time = >1 h; excessive blood loss = 10-volume % decrease in hematocrit; NS = not significant.
[b]Number with percentage in parentheses.
[c]Fisher's exact probability test.

Thirty-three (33%) of the 99 open wounds had bacterial colonization, and 12 other wounds were clinically infected because of cellulitis or purulent drainage but no bacteria were isolated. *Staphylococcus epidermidis* was present in 19 (58%) of open wounds, *Staphylococcus aureus* in 7 (21%), with *Streptococcus pyogenes*, *Streptococcus agalactiae*, Enterococci, gamma hemolytic streptococci, *Coryne-*

**TABLE 3. Preoperative pregnancy demographics in 99 women with wound disruption compared with 1,310 women without this complication[a]**

| Demographic factor | Disrupted (N = 99) | Intact (N = 1,310) | Comparison |
|---|---|---|---|
| Age (years) | 24 ± 6[b] | 25 ± 6[b] | NS |
| Gravidity | 3 ± 2 | 3 ± 2 | NS |
| Parity | 2 ± 1 | 2 ± 1 | NS |
| Obesity | 32 ± 7 | 20 ± 6 | <0.001 |
| Operative time (min) | 42 ± 18 | 48 ± 18 | NS |
| Rupture of membranes (h) | 8 ± 21 | 6 ± 22 | NS |
| Race | | | |
| Hispanic | 38 (38%) | 595 (45%)[c] | NS |
| Black | 43 (43%) | 407 (31%) | NS |
| White | 16 (16%) | 278 (21%) | NS |
| Other | 2 (2%) | 30 (2%) | NS[d] |
| Labor | 68 (68%) | 751 (57%) | NS |
| Prolonged rupture of membranes | 24 (24%) | 169 (12%) | 0.002 |
| Diabetes | 4 (4%) | 51 (4%) | NS[d] |
| Amnionitis | 13 (13%) | 97 (7%) | 0.041 |

[a]Differences in the mean compared with Student's t-test; differences in the frequency compared with chi square; obesity = Quetelet's index value >25 [weight (kg)/height$^2$ (m)]; prolonged rupture of membranes = >1 h; amnionitis = fever ≥38°C and/or foul-smelling infant at delivery; NS = not significant.
[b]Mean ± SD.
[c]Number with percentage in parentheses.
[d]Fisher's exact probability test.

*bacterium* sp., *Pseudomonas* sp., *Acinetobacter* sp., *Escherichia coli, Proteus mirabilis,* and *Citrobacter* sp. comprising the remaining organisms. Sixteen women had disrupted wounds that were colonized with 2–4 different organisms.

Logistic regression analysis was used to determine those potential preoperative factors significantly associated with superficial wound disruption. Shown in Table 3 are selected preoperative demographic factors in the 99 women with superficial wound disruption compared with the 1,310 women with intact wounds. Obesity ($P < 0.001$), prolonged membrane rupture ($P = 0.002$), and amnionitis in labor ($P = 0.04$) were significantly increased in women subsequently developing superficial wound disruption. Other factors such as prolonged operative time, excessive blood loss, and diabetes were not predictive of wound disruption. Shown in Table 4 are intraoperative and puerperal characteristics in those women with wound disruptions compared with those with intact wounds. Unsutured Scarpa's and Camper's fascia ($P = 0.03$), endometritis ($P = 0.001$), and cesarean sections performed for dystocia ($P = 0.03$) were significantly associated with wound disruption, while no association was found with excessive blood loss or prolonged operative time.

### DISCUSSION

We found the usual forerunners of wound disruption in our patient population but they were inconsistent in predicting wound complications. Obesity and preexisting operative site infection, i.e., amnionitis, increased the risk for development of wound complication, similar to the findings of other investigators.[3] Prolonged operative time, excessive blood loss at surgery, and diabetes have also been reported to be associated with an increased risk of wound disruption in some studies[2,8,10] but were not predictors in the present investigation.

A clean wound is one in which the alimentary, respiratory, and genitourinary tracts were not entered and in which aseptic technique was maintained and no apparent inflammation was present.[14] A clean-contaminated wound is one in which a minor break in technique occurred or in which the respiratory, gastrointestinal, or genitourinary tracts were entered but with no significant spillage.[14] Wound disruption has been reported to occur in 2–4% of clean operations and 10–20% of those classified as clean-contaminated.[1,2,10] Cesarean sections are usually classified as clean or clean-contaminated procedures and are associated with a widely divergent frequency of wound complications. The clinical wound infection rate after cesarean section

**TABLE 4. Intraoperative and puerperal characteristics in 99 women with wound disruption compared with 1,310 women without this complication[a]**

| Characteristic | Disrupted (N = 99) | Intact (N = 1,310) | Comparison |
|---|---|---|---|
| Indication for cesarean section | | | |
| Repeat | 40 (40%)[b] | 586 (45%)[b] | NS |
| Dystocia | 35 (35%) | 308 (24%) | 0.03 |
| Fetal distress | 13 (13%) | 187 (14%) | NS |
| Abnormal presentation | 9 (9%) | 202 (15%) | NS |
| Other | 2 (2%) | 27 (2%) | NS[c] |
| Prolonged operative time | 11 (11%) | 216 (16%) | NS |
| Excessive blood loss | 5 (5%) | 61 (4%) | NS[c] |
| Endometritis | 39 (39%) | 296 (22%) | <0.001 |
| Fascial dehiscence | 3 (3%) | 0 (0%) | NS[c] |
| Subcutaneous suture | 40 (5%) | 59 (8%) | 0.03 |

[a]Frequencies compared with chi square; prolonged operative time = >1 h; excessive blood loss = >10-volume % decrease in hematocrit; NS = not significant.
[b]Number with percentage in parentheses.
[c]Fisher's exact probability test.

ranges from 4.8 to 14%,[1–5,15] while 89% of disrupted wounds were colonized with pathogens.[6]

Fascial dehiscence has been reported in 0.6–12.5% of all disrupted cesarean wounds,[4,6,9,15,16] resulting in a 2–3% maternal mortality.[9,16] Fascial dehiscence occurred in only 0.2% (N = 3) of our study population. All 3 patients with fascial dehiscence were in the group who did not have suture closure of their subcutaneous tissues.

In our population, only obesity, endometritis, and non-sutured closure of the subcutaneous tissues were significantly predictive of wound disruption by logistic regression. Although it does not seem possible to reduce patient obesity at the time of cesarean section, the frequency of metritis can be reduced by prophylactic antibiotics. Importantly, closure of Scarpa's and Camper's fascia during cesarean section (in patients with a vertical skin incision) with interrupted 000 plain catgut suture is easily and rapidly performed with marked benefit to the patient, i.e., less dehiscence. These results may not apply to patients with a pfannenstiel incision, since the rate of dehiscence is significantly lower than with the vertical incision. There were only 5 (2.2%) dehiscences in patients with this latter incision. We conclude that there is significant benefit in approximating Scarpa's and Camper's fascia with suture at the time of cesarean section when surgical staples are used to approximate the skin.

### REFERENCES

1. Ortona L, Federico G, Fantoni M, et al.: A study on the incidence of postoperative infections and surgical sepsis in a university hospital. Infect Control 8:320–324, 1987.
2. Cruse PJE, Foord R: A five-year prospective study of 23,649 surgical wounds. Arch Surg 107:206–210, 1973.
3. Pelle H, Jepson OB, Larsen SO, et al.: Wound infection after cesarean section. Infect Control 7:456–461, 1986.
4. Gibbs RS, Blanco JD, St. Clair PJ: A case control study of wound abscess after cesarean section. Obstet Gynecol 62:498–501, 1983.
5. Moir-Bussy BR, Hutton RM, Thompson JR: Wound infection after cesarean section. J Hosp Infect 5:359–370, 1984.
6. Emmons SL, Krohn M, Jackson M, et al.: Development of wound infections among women undergoing cesarean section. Obstet Gynecol 72:559–564, 1988.
7. Polk HC Jr, Simpson CJ, Simmons BP, Alexander JW: Guidelines for prevention of surgical wound infection. Arch Surg 118:1213–1217, 1983.
8. Schwartz RH: Prevention of wound infections—Changing perceptions. Infect Med Dis Lett Obstet Gynecol 12:34–36, 1990.
9. Helmkamp BF: Abdominal wound dehiscence. Am J Obstet Gynecol 128:803–807, 1977.
10. Charles D: Historical perceptions of wound infections. Infect Med Dis Lett Obstet Gynecol 12:28–33, 1990.
11. Mead PB: Abdominal wound infections. In Schaefer G, Graber EA (eds): Complications in Obstetrics and Gynecologic Surgery. Hagerstown: Harper & Row, chap 10, 1991.
12. Cruse PJ, Foord R: The epidemiology of wound infection. Surg Clin North Am 60:27–41, 1980.
13. Roche AF, Seirvogel RM, Chumlea WC, Webb P: Grading body fatness from limited anthropometric data. Am J Clin Nutr 34:2831–2838, 1981.

14. Olson M, O'Connor M, Schwartz ML: Surgical wound infections. A 5-year prospective study of 20,193 wounds at the Minneapolis VA Medical Center. Ann Surg 199: 253–259, 1984.
15. Poole GV Jr: Mechanical factors in the abdominal wound closure: The prevention of fascial dehiscence. Surgery 97:631–639, 1985.
16. Mowat J, Bonnar J: Abdominal wound dehiscence after caesarean section. Br Med J 2:256–257, 1971.
